# Atomic-Level Insights from SWAXS: Quantifying Uncertainty in Biological Models

**DOI:** 10.1063/4.0000826

**Published:** 2025-10-27

**Authors:** Patrick K. Oduro, Jitendra Singh, Sarah Chamberlain, Thomas D. Grant

**Affiliations:** SUNY University at Buffalo, Buffalo, New York, USA

## Abstract

Small- and wide-angle X-ray scattering (SWAXS) is a powerful technique for investigating the shape and dynamics of biological macromolecules in solution. Despite its growing popularity, the field has lacked standardized criteria for evaluating data precision and model accuracy, undermining confidence in results and limiting wider adoption. To address this critical gap, we introduce a suite of objective assessment metrics based on Shannon information theory that quantitatively evaluate both data precision and model-data agreement. Our approach provides three key advances: (1) accurate quantification of the amount and precision of information in a SWAXS profile that enables objective comparison between datasets; (2) a data quality-aware measure of model-data fit that provides more trustworthy structural assessments; and (3) a novel statistical framework that leverages conformational sampling to estimate how far a model deviates from the true structure based on the data. Importantly, our method identifies which regions of structural models are most reliably determined with per-atom coordinate uncertainty estimates. Validation across diverse benchmark and simulated datasets demonstrates that these metrics are robust, intuitive, and provide meaningful assessments of SWAXS models, which are complimentary to existing guidelines for publication. This integrated framework transforms how structural data from SWAXS experiments can be interpreted, offering the standardized evaluation criteria long needed by the scientific community and enhancing confidence in SWAXS-based structural biology.

